# Antibiotic Treatment for First Episode of Acute Otitis Media Is Not Associated with Future Recurrences

**DOI:** 10.1371/journal.pone.0160560

**Published:** 2016-09-15

**Authors:** Marthe te Molder, Marieke L. A. de Hoog, Cuno S. P. M. Uiterwaal, Cornelis K. van der Ent, Henriette A. Smit, Anne G. M. Schilder, Roger A. M. J. Damoiseaux, Roderick P. Venekamp

**Affiliations:** 1 Julius Center for Health Sciences and Primary Care, University Medical Center Utrecht, Utrecht, The Netherlands; 2 Department of Pediatric Pulmonology, Wilhelmina Children's Hospital, University Medical Center Utrecht, Utrecht, The Netherlands; 3 Department of Otorhinolaryngology, University Medical Center Utrecht, Utrecht, The Netherlands; 4 Ear Institute, University College London, London, United Kingdom; University of Chieti, ITALY

## Abstract

**Objective:**

Antibiotic treatment of acute otitis media (AOM) has been suggested to increase the risk of future AOM episodes by causing unfavorable shifts in microbial flora. Because current evidence on this topic is inconclusive and long-term follow-up data are scarce, we wanted to estimate the effect of antibiotic treatment for a first AOM episode occurring during infancy on AOM recurrences and AOM-related health care utilization later in life.

**Methods:**

We obtained demographic information and risk factors from data of the Wheezing Illnesses Study Leidsche Rijn, a prospective birth cohort study in which all healthy newborns born in Leidsche Rijn (between 2001 and 2012), The Netherlands, were enrolled. These data were linked to children’s primary care electronic health records up to the age of four. Children with at least one family physician-diagnosed AOM episode before the age of two were included in analyses. The exposure of interest was the prescription of oral antibiotics (yes vs no) for a child’s first AOM episode before the age of two years.

**Results:**

848 children were included in analyses and 512 (60%) children were prescribed antibiotics for their first AOM episode. Antibiotic treatment was not associated with an increased risk of total AOM recurrences (adjusted rate ratio: 0.94, 95% CI: 0.78–1.13), recurrent AOM (≥3 episodes in 6 months or ≥4 in one year; adjusted risk ratio: 0.79, 95% CI: 0.57–1.11), or with increased AOM-related health care utilization during children’s first four years of life.

**Conclusions:**

Oral antibiotic treatment of a first AOM episode occurring during infancy does not affect the number of AOM recurrences and AOM-related health care utilization later in life. This information can be used when weighing the pros and cons of various AOM treatment options.

## Introduction

Acute otitis media (AOM) is one of the most common reasons for primary care consultations during early childhood and a prime indication for antibiotic prescriptions.[1] In general, AOM has a favorable natural course with symptoms settling spontaneously within a few days in most children.[2–4] However, serious suppurative complications such as mastoiditis, meningitis and intracranial abscess occur in a small subset of children.[2,5]

Although antibiotics have been shown to reduce AOM symptoms more quickly than placebo[4], AOM practice guidelines[6,7] advice a judicious use of antibiotics in this condition because of its limited effect on preventing suppurative complications [2,5,8] and the potential harms related to its use. Oral antibiotics are associated with an increased risk of systemic side effects such as vomiting, diarrhea and rash[4] and their routine use enhances the risk of antimicrobial resistance.[9] Furthermore, it has been hypothesized that antibiotics put children at risk for future infections.[10,11] Several studies showed that antibiotics affect the nasopharyngeal microbial flora[12], which in turn has been suggested to enhance overgrowth and spread of potential pathogens.[10,11,13] Moreover, recent studies showed that biofilm formation occurred 2–5 times faster in patients treated with antibiotics compared with those that did not receive antibiotic treatment.[14] Since the biofilm serves as a bacterial reservoir in which bacteria are 1000 times more resistant to the action of antibiotics than their planktonic counterparts, biofilm formation has been associated with an increased risk of recurrent infections.[15]

Thus far, only one study assessed the long-term (>12 months) impact of antibiotic treatment on future AOM episodes.[16] This placebo-controlled trial showed that treatment with oral antibiotics of AOM at young age was associated with a 50% increase in the risk of AOM recurrences at long term follow-up.[16] However, the response rate was only 70% and data were collected retrospectively. Other randomized controlled trials, comparing antibiotics with placebo or watchful waiting in children with AOM, did not find any differences in AOM recurrences at short term follow-up (up to 12 months).[4,17]

More research is needed to determine whether antibiotic treatment for AOM during infancy puts children at risk of developing further AOM recurrences later in life. We linked our large, prospective birth cohort data to children’s primary care electronic health records to estimate the long-term effects of antibiotic treatment of a first AOM episode occurring during infancy on AOM occurrence and AOM-related health care utilization during children’s first four years of life.

## Methods

### Study design and participants

Data were collected as part of the WHeezing Illnesses STudy LEidsche Rijn (WHISTLER), a prospective birth cohort study. Parents of healthy newborns, born between December 2001 and December 2012, living in Leidsche Rijn (district of Utrecht, The Netherlands) were invited to participate within three weeks after their child’s birth. Exclusion criteria were gestational age <36 weeks, major congenital abnormalities and neonatal respiratory disease. Prior to enrollment, written informed consent was obtained from parents of participating children. Study design and rationale of WHISTLER are described in detail elsewhere.[18] WHISTLER was approved by the pediatric medical ethics committee of the University Medical Center Utrecht (project approval number 01/176).

### Data collection

For this study we used (i) baseline data on prenatal risk factors and parental characteristics, and (ii) prospectively collected data on postnatal risk factors from monthly questionnaires during the child’s first year of life. Additionally, (iii) four years of follow-up data were extracted from primary care electronic health records for all participating children with a family physician practicing within the Leidsche Rijn research district, using uniform codes for diagnoses and treatments according to the International Classification of Primary Care (ICPC) and Anatomical Therapeutical Chemical (ATC) coding systems. We extracted data on AOM (ICPC H71) episodes, AOM-related health care utilization including consultations, antibiotic prescriptions and specialist referrals, and data on all-cause antibiotic prescriptions prior to the first AOM episode.

### Exposure

The exposure variable of interest was the prescription of oral antibiotics (ATC code J01, yes vs no) for a child’s first AOM episode occurring before two years of age and presented to the family physician. ‘No oral antibiotics’ was chosen as reference category.

### Outcome

The primary outcome was the total number of AOM recurrences during children’s first four years of life seen by a family physician. A new episode of AOM (ICPC H71) was documented after an interval of at least 28 days without AOM-related consultations. Secondary outcomes included (i) the occurrence of recurrent AOM (yes vs no) defined as three or more episodes of AOM within six months, or four episodes within 12 months [7], and (ii) AOM-related health care utilization (consultations, antibiotic prescriptions and specialist referrals) during the first four years of life.

### Confounders

We considered the following characteristics as confounders: gender, season of birth, parental education level, duration of exclusive breastfeeding, number of older siblings, household-smoking, daycare attendance, age of first AOM episode and number of oral antibiotics prior to the first AOM episode (irrespective of indication).

Parental education level, as a proxy for socio-economic status, was based on the highest completed education of the child’s parents. If at least one of the parents was highly educated (professional degree or bachelor’s degree or higher), education level was defined as ‘high’. Otherwise it was defined as ‘middle/low’. Household-smoking was categorized in number of months of the first year of life of household-smoking (of at least half a day per week). Daycare attendance was based on the age of first daycare visit (of at least half a day per week).

### Statistical analyses

Because of missing values in parental- and child-related factors, we first imputed missing values using the multiple imputation by chained equations (MICE) procedure in SPSS.[19] We created 10 imputed data sets. A pooled analysis was performed to give a single mean estimate and adjusted standard errors according to the Rubin’s rule.[20]

Next, we compared characteristics of children who did and did not receive oral antibiotics for their first AOM episode using Chi-squared tests, Mann-Whitney U tests and independent samples T-tests.

We then studied the association between oral antibiotic treatment (yes vs no) for a child’s first AOM episode occurring during the first two years of life and our outcome variables of interest. Associations between antibiotic treatment and count outcomes (total number of AOM recurrences, number of AOM-related consultations and antibiotic prescriptions) were calculated using negative binomial regression analyses. These regression coefficients reflect rate ratios. Associations between antibiotic treatment and dichotomous outcomes (incidence of recurrent AOM, incidence of AOM-related specialist referrals) were estimated using Poisson regression analyses with robust standard errors[21]. The regression coefficients derived from these analyses reflect risk ratios.

In all analyses, follow-up duration was used as the offset variable to indicate exposure time. Follow-up duration was estimated as the time in months from the first family physician diagnosed AOM episode until the last known date of follow-up in primary care, or until children’s fourth birthday.

All models were adjusted for potential confounders and all statistical analyses were performed with SPSS version 21.0 (IBM SPSS, Armonk, NY, USA).

## Results

**Fig 1:**
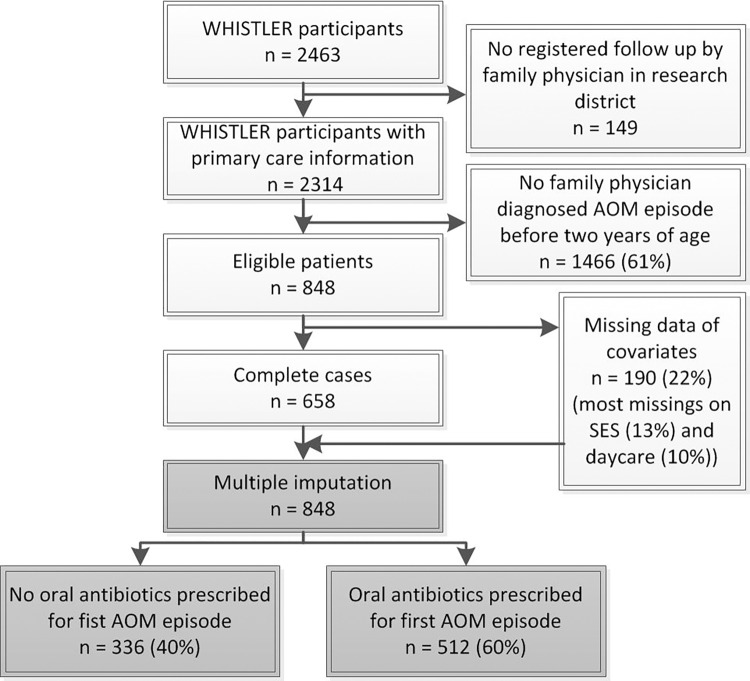
Flow chart of study population Abbreviations: AOM, acute otitis media. SES, socio-economic status.

During the WHISTLER recruitment period (December 2001 to December 2012), parents of 9236 children were invited of whom 2463 children were enrolled after obtaining full written informed consent. 2314 (94%) had a family physician inside the research district and at least one day of follow up, which allowed us to extract relevant primary care health electronic records data. 848 of 2314 children (37%) experienced a family physician diagnosed AOM episode before the age of two years (Fig 1). Of those, 190 (22%) had missing confounders data which were imputed using multiple imputation techniques.

Baseline characteristics of the 848 included children are summarized in Table 1. 512 (60%) children were prescribed oral antibiotics for their first AOM episode before the age of two years. Children receiving antibiotics were significantly older at the time of first AOM episode (mean age 11.3 (SD 5.2) vs 9.9 months (SD 5.1), *p*<0.001) than those in which antibiotics were not prescribed. Half of the children (60/120) having their first AOM episode before the age of six months were treated with antibiotics. Moreover, the number of older siblings was significantly higher in children receiving antibiotics than in those who did not receive antibiotics (median number of siblings 1 (IQR 0–1) vs 0 (IQR 0–1), p = 0.02).

**Table 1:** Demographic and risk factors at enrollment of completed cases.

Characteristics of study population	First episode of AOM treated with oral antibiotics		
	No (n = 336)	Yes (n = 512)	*p*-value
Gender, n (%)			
Male	166 (49.4%)	276 (53.9%)	0.20
Female	170 (50.6%)	236 (46.1%)	
Parental education level, n (%)			
High educated	258 (76.8%)	395 (77.1%)	0.90
Middle/low educated	78 (23.2%)	117 (22.9%)	
Season of birth, n (%)			
Spring	80 (23.8%)	148 (28.9%)	0.12
Summer	92 (27.4%)	124 (24.2%)	
Fall	67 (19.9%)	119 (23.2%)	
Winter	97 (28.9%)	121 (23.6%)	
Number of siblings, median (IQR)	0 (0–1)	1 (0–1)	0.02
Presence of older siblings, n (%)			
0–4 years old	161 (82.1%)	282 (79.4%)	0.48
5–8 years old	26 (13.3%)	60 (16.9%)	
9–12 years old	9 (4.6%)	13 (3.7%)	
Duration of exclusive breastfeeding, n (%)			
No or only partial breastfeeding	59 (17.6%)	117 (22.9%)	0.37
<3 months	160 (47.6%)	224 (43.8%)	
3–6 months	79 (23.5%)	111 (21.7%)	
>6 months	38 (11.3%)	60 (11.7%)	
Household-smoking during first year, n (%)			
No smoking	261 (77.7%)	418 (81.6%)	0.36
1–5 months of smoking	58 (17.3%)	74 (14.5%)	
≥ 6 months of smoking	17 (5.1%)	20 (3.9%)	
Age at entry to daycare, n (%)			
<3 months	43 (12.8%)	65 (12.7%)	0.96
3–5 months	196 (58.3%)	297 (58.0%)	
6–12 months	40 (11.9%)	67 (13.1%)	
No daycare attendance during first year of life	57 (17.0%)	83 (16.2%)	
Age of first AOM episode in months, mean (SD)	9.9 (5.1)	11.3 (5.2)	<0.001
Number of prescribed oral antibiotics, prior to the first AOM episode, n (%)			
0	298 (88.7%)	435 (85.0%)	0.07
1	38 (11.3%)	71 (13.9%)	
≥2	0 (0%)	6 (1.2%)	

Abbreviations: AOM, acute otitis media. SD, standard deviation. IQR, interquartile range.

Of the children who received antibiotics for their first AOM episode, 60.7% had one or more AOM recurrences after the first AOM episode in the first four years of life (range 1–10 recurrences) and 12.3% developed recurrent AOM. Of the children not treated with antibiotics, 62.8% had one or more AOM recurrences (range 1–11 recurrences) and 16.7% developed recurrent AOM. Antibiotic treatment of the first AOM episode was neither associated with an increased risk of total AOM recurrences (adjusted rate ratio: 0.94, 95% CI: 0.78–1.13) and the occurrence of recurrent AOM (adjusted risk ratio: 0.79, 95% CI: 0.57–1.11) (Table 2), nor with the number of AOM-related consultations (adjusted rate ratio: 0.88, 95% CI: 0.74–1.03), antibiotic prescriptions (adjusted rate ratio: 0.93, 95% CI: 0.75–1.16) and specialist referrals (adjusted risk ratio: 0.75 (0.51–1.10)) (Table 3).

**Table 2:** Risk of AOM recurrences during the first four years of life by treatment of first episode.

	Antibiotic treatment for first AOM episode	
	**Crude rate ratio (95% CI)**	**Adjusted rate ratio**^**a**^ **(95% CI)**
**Total number of AOM recurrences**	0.87 (0.73–1.04)	0.94 (0.78–1.13)
	**Crude risk ratio (95% CI)**	**Adjusted risk ratio**^**a**^ **(95% CI)**
**Recurrent AOM**	0.77 (0.55–1.07)	0.79 (0.57–1.11)

In all analysis follow-up duration was used as the offset variable.

^a^ Adjusted for confounders: gender, season of birth, parental education level, duration of exclusive breastfeeding, number of older siblings, household-smoking, daycare attendance, age of first AOM episode and number of oral antibiotics prior to the first AOM episode (irrespective of indication).

Abbreviations: AOM, acute otitis media. CI, confidence interval.

**Table 3:** Use of health care resources during the first four years of life by treatment of first episode.

	Antibiotic treatment for first AOM episode	
	**Crude rate ratio (95% CI)**	**Adjusted rate ratio**^**a**^ **(95% CI)**
**Total number of consultations**	0.79 (0.67–0.93)	0.88 (0.74–1.03)
**Total number of prescribed oral antibiotics**	0.86 (0.70–1.05)	0.93 (0.75–1.16)
	**Crude risk ratio (95% CI)**	**Adjusted risk ratio**^**a**^ **(95% CI)**
**Incidence of specialist referral**	0.81 (0.55–1.17)	0.75 (0.51–1.10)

In all analysis follow-up duration was used as the offset variable.

^**a**^ Adjusted for confounders: gender, season of birth, parental education level, duration of exclusive breastfeeding, number of older siblings, household-smoking, daycare attendance, age of first AOM episode and number of oral antibiotics prior to the first AOM episode (irrespective of indication).

Abbreviations: AOM, acute otitis media. CI, confidence interval.

## Discussion

Oral antibiotic treatment for a first AOM episode occurring during infancy did not affect the total number of AOM recurrences, the occurrence of recurrent AOM and AOM-related health care utilization later in life.

Our findings are in agreement with results of randomized controlled trials assessing the short-term (1.5–12 months) impact of oral antibiotic treatment on AOM recurrences.[4,17] Furthermore, our findings are in line with placebo-controlled trials results of other common respiratory infectious diseases including tonsillitis[22] and acute sinusitis[23,24], showing no impact of antibiotics on recurrence rates. However, our results are conflicting with the only long-term follow-up study performed thus far. This double-blind, placebo-controlled randomized trial of Bezáková et al. found an increased risk of AOM recurrences up to 3.5 years after randomization in children treated with antibiotics.[16] This latter study has, however, some important limitations which may hamper the interpretation of the results: data on the outcomes were collected retrospectively by a parental reported questionnaire with a response rate of only 70% and this is likely to introduce a substantial risk of recall and attrition bias.

When interpreting our results, it should be taken into account that amoxicillin 40mg/kg is the first line antibiotic in AOM in the Netherlands; a safe and efficient dose according to current antibiotic resistance patterns in this country.[6] Bezáková et al. used the same dosage in their trial.[16] However, in many other countries, including the US, a higher dose of amoxicillin (up to 90mg/kg) is recommended.[7] Further research is needed to determine whether the effect of oral antibiotics on AOM recurrences is dose-dependent.

In our study, 848 of 2314 participants (37%) had at least one AOM episode by 2 years which is relatively low. There are, however, various explanations for this. First, we included all newborns who had at least one day of follow-up by their family physician. A substantial number of children (n = 242) were followed for less than two years and were therefore less likely to have an AOM episode recorded by their family physician during their first two year of life. Second, we focused on family physician diagnosed AOM. Previous research, however, indicated that parents consult their family physician with their child in case of AOM symptoms only in about half of the episodes.[25] As such, the community incidence of AOM is likely to be much higher than the estimate found in our study.

We found children receiving antibiotics to be significantly older at the time of first AOM episode than children who did not receive antibiotics. Although current guidance recommends immediate antibiotics in all children aged below six months with AOM, only half of our children were actually treated with antibiotics. Further studies are warranted to confirm this finding, but this suggests that future studies on guideline adherence should not only focus on overtreatment with antibiotics but also on under treatment in specific at-risk groups.

The major strengths of our study are the large sample size and the prospective, observational data collection. Moreover, results were adjusted for a large amount of well-established confounders.

Still, some methodological limitations deserve further attention. First, although family physicians were trained to use of the ICPC coding system and strict diagnostic and treatment criteria are provided by the Dutch family physician AOM practice guideline, we cannot rule out that variability between individual family physicians and physician-related factors (years of experience, available time per patient) influenced coding and management strategies.[26] However, this potential variability may lead to non-differential misclassification but is unlikely to produce biased estimates of the associations.

Second, our study is an observational study so antibiotics were not randomly prescribed to children with AOM. Children receiving antibiotics for their first AOM episode may have been more severely ill and may have a worse prognosis compared with those who did not receive antibiotics, resulting in confounding by indication. This may have led to an overestimation of the number of AOM recurrences in children who were treated with antibiotics for their first AOM episode. Although many confounders were taken into account to minimize the risk of confounding, residual confounding cannot be entirely excluded. For example, we had no information of pacifier use which has been previously associated with an increased risk of recurrent AOM.[27]

Third, we only measured family physician diagnosed AOM episodes which is, as mentioned previously, likely to underestimate the burden of AOM in the community.[25] It is likely that not all parents of participating children did consult their family physician when their child suffered from AOM symptoms. This would not necessarily bias our results if health seeking behavior of parents of children with AOM in both exposure groups would be similar. However, it has been shown that AOM-related antibiotic prescriptions are associated with higher doctor consultation rates in case of recurrent episodes.[28] Our study results may therefore overestimate the number of AOM recurrences in children who did receive oral antibiotics for their first AOM episode.

Fourth, we collected ATC codes to measure exposure status (oral antibiotics, yes vs no). However, prescribing antibiotics does not necessarily imply that the child actually took the prescribed drug. A previous study on the use of antibiotics among ambulatory pediatric patients reported a compliance rate of 78.9%.[29] It is unlikely that this non-compliance introduced a differential bias, but it may have caused non-differential misclassification of the exposure. As such, it may have weakened the differences between the two groups.

If those last three limitations would have affected our results, the “true” risk of AOM recurrences could actually be lower after antibiotic treatment, and thereby be in the opposite direction of the previously mentioned results of Bezáková et al.[16]

Concluding, antibiotic treatment of a first AOM episode occurring during infancy is neither associated with an increased risk of AOM recurrences, nor with increased AOM-related health care utilization during children’s first four years of life. This information can be used when weighing the pros and cons of various treatment options in children experiencing their first AOM during life infancy.

## References

[1] Gulliford M, Latinovic R, Charlton J, Little P, Van Staa T, Ashworth M. Selective decrease in consultations and antibiotic prescribing for acute respiratory tract infections in UK primary care up to 2006. J Public Health (Oxf). 2009; 31: 512–520. 19734168 10.1093/pubmed/fdp081 PMC2781723

[2] Rosenfeld RM, Kay D. Natural history of untreated otitis media. Laryngoscope 2003; 113:1645–57. 14520089 10.1097/00005537-200310000-00004

[3] Thompson M, Vodicka TA, Blair PS, Buckley DI, Heneghan C, Hay D: TARGET Programme Team. Duration of symptoms of respiratory tract infections in children: systematic review. BMJ. 2013;347(f7027). 10.1136/bmj.f7027 PMC3898587 24335668

[4] Venekamp RP, Sanders SL, Glasziou PP, Del Mar CB, Rovers MM. Antibiotics for acute otitis media in children. Cochrane Database Syst Rev. 2015;:CD000219 10.1002/14651858.CD000219.pub4 11034677

[5] Thompson PL, Gilbert RE, Long PF, Saxena S, Sharland M, Wong IC. Effect of antibiotics for otitis media on mastoiditis in children: a retrospective cohort study using the United Kingdom general practice research database. Pediatrics. 2009;123:424–430. 10.1542/peds.2007-3349 19171605

[6] Damoiseaux RAMJ, Venekamp RP, Eekhof JAH, Bennebroek Gravenhorst FM, Schoch AG, Burgers JS et al Otitis media acuta bij kinderen (derde herziening) [Translated from Dutch: Acute otitis media in children (third revision)]. Huisarts wet. 2014;57:648.

[7] American Academy of Pediatrics, American Academy of Family Physicians. Clinical practice guidelines: diagnosis and management of acute otitis media. Pediatrics. 2013;131:964–99.

[8] Grossman Z, Zehavi Y, Leibovitz E, Grisaru-Soen G, Shachor Meyouhas Y, Kassis I et al Severe Acute Mastoiditis Admission is Not Related to Delayed Antibiotic Treatment for Antecedent Acute Otitis Media. Pediatr Infect Dis J. 2016;35:162–5. 10.1097/INF.0000000000000951 26461229

[9] Costelloe C, Metcalfe C, Lovering A, Mant D, Hay AD. Effect of antibiotic prescribing in primary care on antimicrobial resistance in individual patients: systematic review and meta-analysis. BMJ. 2010;340:C2096 10.1136/bmj.c2096 20483949

[10] De Steenhuijsen Piters WAA, Sanders EAM, Bogaert D. The role of the local microbial ecosystem in respiratory health and disease. Philos Trans R Soc Lond B Biol Sci. 2015;370:20140294 10.1098/rstb.2014.0294 26150660 PMC4528492

[11] Brook I, Gober AE. Antimicrobial resistance in the nasopharyngeal flora of children with acute otitis media and otitis media recurring after amoxicillin therapy. J Med Microbiol. 2005;54:83–85. 15591260 10.1099/jmm.0.45819-0

[12] Cohen R, Bingen E, Varon E, de La Rocque F, Brahimi N, Levy C et al Change in nasopharyngeal carriage of Streptococcus pneumoniae resulting from antibiotic therapy for acute otitis media in children. Pediatr Infect Dis J. 1997;16:555–560. 9194104 10.1097/00006454-199706000-00004

[13] Ghaffar F, Muniz LS, Katz K, Smith JL, Shouse T, Davis P et al Effects of large dosages of amoxicillin/clavulanate or azithromycin on nasopharyngeal carriage of Streptococcus pneumoniae, Haemophilus influenzae, nonpneumococcal α-hemolytic strept, and Staphylococcus aureus in children with acute otitis media. Clin Infect Dis. 2002;34:1301–1309. 11981724 10.1086/340054

[14] Balcázar JL, Subirats J, Borrego CM. The role of biofilms as environmental reservoirs of antibiotic resistance. Front Microbiol. 2015; 6:1216 10.3389/fmicb.2015.01216 26583011 PMC4628128

[15] Bakaletz LO. Bacterial biofilms in the upper airway—evidence for role in pathology and implications for treatment of otitis media. Paediatr Respir Rev. 2012;13:154–9. 10.1016/j.prrv.2012.03.001 22726871 PMC3509202

[16] Bezáková N, Damoiseaux RA, Hoes AW, Schilder AG, Rovers MM. Recurrence up to 3.5 years after antibiotic treatment of acute otitis media in very young Dutch children: survey of trial participants. BMJ. 2009;339:b525. 10.1136/bmj.b2525 PMC3272660 19567910

[17] Damoiseaux RA, Rovers MM, Van Balen FA, Hoes AW, de Melker RA. Long-term prognosis of acute otitis media in infancy: determinants of recurrent acute otitis media and persistent middle ear effusion. Fam Pract. 2006;23:40–45. 16107490 10.1093/fampra/cmi083

[18] Katier N, Uiterwaal CSPM, De Jong BM, Kimpen JL, Verheij TJ, Grobbee DE et al The wheezing illnesses study Leidsche Rijn (WHISTLER): Rationale and design. Eur J Epidemiol. 2004;19:895–903. 15499901 10.1023/B:EJEP.0000040530.98310.0c PMC7087709

[19] Donders AR, van der Heijden GJ, Stijnen T, Moons KG. Review: a gentle introduction to imputation of missing values. J Clin Epidemiol. 2006;59:1087–1091. 16980149 10.1016/j.jclinepi.2006.01.014

[20] Rubin DB. Multiple Imputation for Non-Response in Surveys. In: New York: John Wiley; 1987.

[21] Knol MJ, Le Cessie S, Algra A, Vandenbroucke JP, Groenwold RHH. Overestimation of risk ratios by odds ratios in trials and cohort studies: alternatives to logistic regression. Can Med Assoc J. 2012;184:895–899. 22158397 10.1503/cmaj.101715 PMC3348192

[22] Zwart S. Penicillin for acute sore throat: randomised double blind trial of seven days versus three days treatment or placebo in adults. BMJ. 2000;320:150–154. 10634735 10.1136/bmj.320.7228.150 PMC27262

[23] Van Buchem FL, Knottnerus JA, Schrijnemaekers VJ, Peeters MF. Primary-care-based randomised placebo-controlled trial of antibiotic treatment in acute maxillary sinusitis. Lancet. 1997;349:683–7. 9078199 10.1016/s0140-6736(96)07585-x

[24] Garbutt JM, Banister C, Spitznagel E, Piccirillo JF. Amoxicillin for acute rhinosinusitis: a randomized controlled trial. JAMA. 2012;307:685–92. 10.1001/jama.2012.138 22337680 PMC3667493

[25] Fortanier AC, Venekamp RP, de Hoog ML, Uiterwaal CS, van der Gugten AC, van der Ent CK et al Parent-reported symptoms of acute otitis media during the first year of life: what is beneath the surface? PLoS One. 2015;10:e0121572 10.1371/journal.pone.0121572 25849847 PMC4388588

[26] Akkerman AE, Kuyvenhoven MM, van der Wouden JC, Verheij TJ. Prescribing antibiotics for respiratory tract infections by GPs: management and prescriber characteristics. Br J Gen Pract. 2005;55:114–8. 15720932 PMC1463185

[27] Rovers MM, Numans ME, Langenback E, Grobbee DE, Verheij TJ, Schilder AG. Is pacifier use a risk factor for acute otitis media? A dynamic cohort study. Fam Pract. 2008;25:233–6. 10.1093/fampra/cmn030 18562333

[28] Williamson I, Benge S, Mullee M, Little P. Consultations for middle ear disease, antibiotic prescribing and risk factors for reattendance: a case-linked cohort study. Br J Gen Pract. 2006;56:170–175. 16536956 PMC1828259

[29] Hoppe JE, Blumenstock G, Grotz W, Selbmann HK. Compliance of German pediatric patients with oral antibiotic therapy: results of a nationwide survey. Pediatr Infect Dis J. 1999;18:1085–91. 10608630 10.1097/00006454-199912000-00012

